# Oxidative stress as a key modulator of cell fate decision in osteoarthritis and osteoporosis: a narrative review

**DOI:** 10.1186/s11658-023-00489-y

**Published:** 2023-09-30

**Authors:** Jana Riegger, Astrid Schoppa, Leonie Ruths, Melanie Haffner-Luntzer, Anita Ignatius

**Affiliations:** 1 Division for Biochemistry of Joint and Connective Tissue Diseases, Department of Orthopedics, Ulm University Medical Center, 89081 Ulm, Germany; 2 Institute of Orthopedic Research and Biomechanics, Ulm University Medical Center, 89081 Ulm, Germany

**Keywords:** Osteoarthritis, Osteoporosis, Senescence, Oxidative stress, ROS, Cell death, Cell fate decision, Mitochondrial dysfunction, Cartilage, Bone

## Abstract

During aging and after traumatic injuries, cartilage and bone cells are exposed to various pathophysiologic mediators, including reactive oxygen species (ROS), damage-associated molecular patterns, and proinflammatory cytokines. This detrimental environment triggers cellular stress and subsequent dysfunction, which not only contributes to the development of associated diseases, that is, osteoporosis and osteoarthritis, but also impairs regenerative processes. To counter ROS-mediated stress and reduce the overall tissue damage, cells possess diverse defense mechanisms. However, cellular antioxidative capacities are limited and thus ROS accumulation can lead to aberrant cell fate decisions, which have adverse effects on cartilage and bone homeostasis. In this narrative review, we address oxidative stress as a major driver of pathophysiologic processes in cartilage and bone, including senescence, misdirected differentiation, cell death, mitochondrial dysfunction, and impaired mitophagy by illustrating the consequences on tissue homeostasis and regeneration. Moreover, we elaborate cellular defense mechanisms, with a particular focus on oxidative stress response and mitophagy, and briefly discuss respective therapeutic strategies to improve cell and tissue protection.

## Background

The steady increase of age-related diseases might be one of the most impactful downsides of our contemporary society. An unhealthy lifestyle, including insufficient exercise, unhealthy food, and mental stress, enhance the risk of comorbidities in age. In addition, cardiovascular disorders and musculoskeletal diseases, including osteoarthritis (OA) and osteoporosis (OP), are considered to be the most common global age-related diseases. While OA is defined as a whole-joint disease, characterized by cartilage destruction, synovial inflammation, severe pain, and immobility, OP is mainly associated with a decrease in bone mass and subsequent compromised bone strength. Both OA and OP are incurable and associated with disability in age, causing high economic costs. As in most degenerative and age-related diseases, oxidative stress has been identified as a crucial driver of the underlying pathogenesis in OA and OP.

In addition to pro-inflammatory cytokines, reactive oxidative species (ROS) are considered to be the most important modulators of cell fate and behavior. The term “ROS”, which will be used as collective term for both ROS and reactive nitrogen species in this review, comprises unstable, thus reactive, molecules such as free radicals [superoxide (·O_2_^−^), hydroxyl radicals (·OH), or nitric oxide (·NO)] and their secondary products, for example, hydrogen peroxide (H_2_O_2_) or peroxynitrite (ONOO^−^).

It has been estimated that approximately 90% of endogenously produced ROS derive from the mitochondrial electron transfer chain due to electron leakage in the course of ATP production [1]. However, ROS can also be generated in other cell organelles, such as the endoplasmic reticulum, peroxisomes, and lysosomes, as well as in the cytoplasm. Various enzymes are involved in endogenous ROS production, including nicotinamide adenine dinucleotide phosphate (NADPH) oxidases (NOXs), cytochrome P450, xanthine oxidase, and NO synthase among others [2].

At physiologic levels, ROS are considered to be second messengers and involved in cellular function maintenance by orchestrating various processes, including proliferation, survival, and differentiation. Moreover, ROS have been associated with cellular communication and immunomodulation, ranging from immune cell recruitment and activation to immunosuppression [3].

The signal transduction of ROS is mainly based on redox-sensitive signaling pathways, such as the nuclear factor-κB (NFκB) pathway as well as the mitogen-activated protein kinases (MAPKs) pathways extracellular signal-regulated kinase 1/2 (Erk1/2), p38, and c-Jun N-terminal kinases (JNK) [4, 5]. By contrast, several cysteine-containing enzymes can be inactivated by means of an NO-dependent modification of thiol groups, termed S-nitrosylation, as reported in the case of caspases as well as several transcription factors, including the NFκB-related proteins p50 and p65, or the subunits of activation protein 1, c-jun, and c-fos [5]. Therefore, ROS can both activate and inhibit redox-sensitive pathways involved in (patho-)physiologic processes in a different manner. In the context of mitogenic, nutrient uptake, or survival signals, epidermal growth factor) and platelet-derived growth factor, for example, were found to transiently increase ROS generation via NOXs to mediate receptor tyrosine phosphorylation and sustain subsequent signal transduction [4].

In the following narrative review, we will elaborate why oxidative stress occurs upon aging and how ROS regulate cell fate and behavior in different situations. During this journey, we will particularly focus on OA and OP. While cartilage is thought to possess only very poor capacity for repair, bone is characterized by a remarkable endogenous regenerative potential. Therefore, pathophysiologic consequences of oxidative stress, such as regulated cell death and senescence, will be predominantly discussed in the context of OA. By contrast, the decisive role of ROS in regenerative processes, including stem cell differentiation, but also consequences of mitochondrial dysfunction, will be addressed in the context of fracture healing and OP, because bone is normally considered to be a highly regenerative tissue.

First, a general introduction into the cellular antioxidant defense system as well as relevant regulators and pathways will be given. Moreover, the disturbance of the antioxidant defense in OA and OP patients will be addressed, including clinical biomarkers of oxidative stress. Afterwards, we will focus on ROS-mediated cell fate decision in OA, comprising regulated chondrocyte death, such as apoptosis, ferroptosis, necroptosis, and pyroptosis, as well as stress-induced premature senescence (SIPS). Subsequently, we will discuss the role of ROS in bone remodeling and fracture healing as well as the consequences of oxidative stress in cell fate regulation in OP. Moreover, we will focus on mitophagy and describe different therapeutic strategies targeting oxidative stress and mitochondrial dysfunction.

## The cellular antioxidant defense system

Disturbance of the fine-tuned ROS balance has detrimental consequences on cells and thus tissue homeostasis. The product of superoxide and nitric oxide, namely peroxynitrite, represents one of the most cytotoxic ROS. Cell toxicity by peroxynitrite is mainly based on its reaction with biomolecules, resulting in lipid peroxidation, S-nitrosylation of glutathione (GSH) and enzymes, deamination of DNA bases, or even DNA single-strand breakage [6].

Some physiologic processes, for example, osteogenic or chondrogenic differentiation, require well-balanced ROS levels. Differentiation is an energy-intensive process associated with enhanced ROS production due to increased mitochondrial activity to satisfy the required ATP demand. At the same time, ROS are essential inducers of cell differentiation by modulating signaling pathways and subsequent transcription. However, both excessive and too low ROS levels impair differentiation [7–9]. Therefore, an intact cellular antioxidant system is required to sustain a physiologic redox homeostasis and cope with excessive ROS levels, thus ensuring cell function and preventing damage.

The cellular antioxidant system consists of enzymatic antioxidants, for example, superoxide dismutases (SODs), catalase (CAT), and glutathione peroxidase (GPX), as well as non-enzymatic ROS scavengers, for example, GSH, ascorbic acid (vitamin C), α-tocopherol (vitamin E), and carotenoids [10, 11].

SODs are a group of antioxidants that are part of the first line antioxidant defense. In mammals, there are three forms of SOD enzymes: cytoplasmic SOD1 (Cu/ZnSOD), mitochondrial SOD2 (MnSOD), and extracellular SOD3 (Cu/ZnSOD) [12]. SODs convert highly reactive superoxide into less harmful H_2_O_2_ [13], which can then be neutralized into oxygen and water by CAT or GPX. Moreover, SOD2-generated H_2_O_2_ serves, for example, as a positive regulator of mitochondrial biogenesis [14, 15]. Elimination of superoxide by SODs prevents cellular damage and maintains mitochondrial function [16], thus protecting against cell death or senescence. It is noteworthy that global as well as a brain-specific knockout of SOD2 leads to neonatal death as observed between 10 and 25 days after birth, depending on the mouse model [17–19].

The GSH system represents another important component of the first line antioxidant defense, comprising GSH, GPX, and glutathione reductase. GSH can act both as a nonenzymatic free ROS scavenger and as a precursor of GPX, which reduces peroxides by using GSH as an electron donor, resulting in oxidized GSH (GSSG) as an end product. The flavoprotein glutathione reductase, in turn, converts GSSG into its reduced form and thus active GSH in a NADPH-consuming reaction, which closes the GSH redox cycle [20].

Different enzymes and pathways are involved in the regulation of cellular antioxidants, thus maintaining the redox homeostasis and promoting cell protection under oxidative stress. In the following paragraphs, we will address some of the most important regulators in more detail.

### Sirtuins and forkhead box O (FoxO) proteins

Sirtuins comprise a family of seven members—SIRT1–7—of cytosolic and mitochondrial nicotinamide adenine dinucleotide (NAD)-dependent deacetylases. They are essential in terms of cellular anti-aging mechanisms, by being involved in DNA repair, antioxidative defense regulation, and immunomodulation. Therefore, sirtuins are considered to be important targets to counteract age-related diseases, including OA and OP [21, 22]. Indeed, sirtuins have been found to maintain tissue anabolism, prevent apoptotic cell death, and attenuate senescence and inflammation in cartilage [23–27] as well as in bone [28, 29]. Sirtuins are important regulators of autophagy and mitochondria-specific autophagy (mitophagy) in chondrocytes and osteoblasts, thus contributing to cellular survival and function [23, 30–32]. Beyond mitophagy, sirtuins, and in particular mitochondrial SIRT3/4/5, are further involved in mitochondrial function maintenance by regulating mitochondrial biogenesis and translation and deacetylation of various enzymes associated with the electron transfer chain, in particular oxidative phosphorylation complexes [33–36]. Overall, deficiencies in cytoplasmic (SIRT1) or mitochondrial (SIRT3) sirtuins were demonstrated to promote OA and OP progression in mice [29, 37–39].

Sirtuin-mediated cell and tissue protective effects are mainly attributed to the modulation of transcription factors, such as p53, NF‐κB, or FoxO 1, 3A, and 4 [25, 28, 40]. Deacetylation of FoxO proteins, for example, results in gene expression of antioxidant proteins, including SOD2, CAT, peroxiredoxins, thioredoxin, and thioredoxin reductase, thus ameliorating ROS accumulation and subsequent cell damage [40].

FoxO activity is negatively regulated by the phosphatidylinositol 3-kinase/Akt pathway, which promotes AKT-dependent phosphorylation and subsequent cytoplasmic retention. JNK or AMP-activated protein kinase (AMPK) signaling, in turn, results in the activation of FoxO proteins, which enables their nuclear translocation and translation of target genes via the FoxO binding consensus domain [41–43]. Both pathways are considered to be stress-responsive and play an important role in ROS defense and cell survival during oxidative stress [44]. FoxO1 and 3 expression was found to be suppressed in aged and OA cartilage derived from both, human donors and experimental murine models, accompanied by enhanced FoxO phosphorylation and thus inactivation of the proteins [45].

### Keap1-Nrf2 pathway

The Keap1-Nrf2 pathway has been considered to be the most important classical regulatory mechanism regarding cellular detoxification and antioxidant defense [46]. Indeed, nuclear factor erythroid 2-related factor 2 (Nrf2) represents a master regulator of antioxidant and cell protective genes, by binding and activating *cis*-acting enhancer antioxidant response element (ARE) sequences [47]. AREs are located in the promoters of genes encoding proteins associated with detoxification and antioxidative defense, for example, SODs, CAT, quinone reductase, glutathione S-transferase, heme oxygenase-1 (HO-1), thioredoxin, and UDP-glucuronyl transferase, as well as the Nrf2 gene itself [46, 48, 49]. Nrf2 activity is constitutively regulated by Kelch-like ECH-associated protein 1 (Keap1), which binds to Nrf2, resulting in its cytosolic retention and the promotion of Cullin 3-mediated ubiquitination and subsequent proteasomal degradation [50]. Upon exposure to oxidative or electrophilic stress, the cysteine-based redox sensors of Keap1 are oxidized, which allows newly translated Nrf2 to translocate into the nucleus, heterodimerize with small Maf proteins, and initiate ARE-dependent transcription [51–53]. In addition to the transcriptional control of antioxidants, the Nrf2 pathway has been described to be involved in mitochondrial homeostasis. Nrf2 was found to induce mitochondrial biogenesis by promoting the transcription of mitochondrial transcription factor A—the key enhancer protein of mitochondrial DNA (mtDNA) replication [54, 55]. Moreover, Nrf2 contributes to mitophagy-mediated elimination of dysfunctional mitochondria by regulating the transcription of PTEN-induced putative kinase 1 (PINK1) [56, 57] and the autophagic adaptor protein sequestosome-1 (p62/SQSTM1) [58]. Interestingly, p62, in turn, has been found to interact with the Nrf2-binding site on Keap1, thus competing with Nrf1. Consequently, elevated p62 levels result in Nrf2 pathway activation, creating a positive feedback loop [58, 59]. Due to its protective role during cellular stress, Nrf2 activation represents a promising therapeutic strategy in acute tissue trauma as well as age-related diseases, comprising neurodegenerative diseases as well as OA and OP [60–64]. Indeed, Nrf2 deficiency was found to induce age-related OP in female mice [65] and OA in different mouse models [66].

### Hypoxia-inducible factor (HIF)-1/2α pathway

Hypoxia-inducible factor-1 α and 2 α (HIF-1/2α) are highly conserved transcription factors, which both heterodimerize with HIF-1β upon hypoxia, forming the transcription factors HIF-1 and HIF-2, respectively. Subsequent binding to the hypoxia responsive element sequence promotes a positive transcriptional response [67]. However, HIF-1 and HIF-2 differ regarding the target genes, as demonstrated in the case of OA, where these transcription factors mediate virtually opposite effects [68].

Under normoxic conditions, the oxygen-dependent degradation domains of the HIF-α subunits are post-translationally hydroxylated by prolyl hydroxylases, which promotes their interaction with the von Hippel-Lindau tumour suppressor protein and induces subsequent ubiquitination and proteasomal degradation [69]. Hydroxylation of specific proline residues and subsequent ubiquitination does not occur under hypoxic conditions, allowing HIF-α subunit accumulation and nuclear translocation. In the nucleus, the HIF-α subunit dimerises with HIF-1β and binds to hypoxia responsive element-containing promoter regions, regulating the transcription of hundreds of target genes [70, 71].

It is understood that crosstalk occurs between the oxygen- and redox-responsive pathways. Indeed, hypoxia leads to mitochondrial ROS accumulation resulting from inefficient electron transfer through the electron transfer chain due to oxygen limitation—the terminal electron acceptor [72]. Mitochondrial ROS, in turn, trigger hypoxia-induced transcription through HIF-1/2a [73, 74]. HIF-1 has been reported to alleviate hypoxia-related mitochondrial ROS generation in different ways. For example, it can act as a regulator of mitophagy by inducing the gene expression of Bcl-2/adenovirus E1B 19-kDa interacting protein, an essential factor in beclin1-dependent autophagy and mitophagy [75], thus promoting cell and tissue protection as well as tissue regeneration after injury, as demonstrated during fracture healing [76]. In detail, mitophagy-driven elimination of dysfunctional mitochondria prevents mitochondrial ROS accumulation, and consequent apoptotic cell death and senescence, as demonstrated in different animal models, including a surgery-induced OA model [75, 77]. By contrast, HIF-1 was observed to be involved in the reduction of mitochondrial cristae, mitochondrial biogenesis, and oxidative stress levels by induction of Hes-related family BHLH transcription factor with YRPW motif, a negative regulator of PINK1, in the context of hepatocellular carcinoma [78].

Interestingly, HIF-1 transcription was found to be enhanced upon oxidative stress, resulting in an induction of different sets of HIF-1 target genes in pulmonary artery and vascular smooth muscle cells [79, 80]. It has been assumed that ROS upregulates HIF-1 transcription via NFκB pathway activation and subsequent binding of p50 and p65 at a newly identified NFκB binding site in the HIF-1 promoter region [79]. As well as this NFκB binding site, additional Nrf2-binding sites, AREs, were found recently in *HIF1*α promoters [81]. Enhanced HIF-1 expression might counteract degradation of the protein and thus allow HIF-1 signaling under mild hypoxia conditions [82].

Beyond the transcriptional level, HIFs have also been demonstrated to be post-translationally regulated by ROS. Indeed, acetylated HIF-1 can be inactivated by the redox-sensing deacetylase SIRT1. During hypoxia, SIRT1 is assumed to be downregulated due to decreased NAD^+^ levels, thus promoting HIF-1α acetylation and its subsequent activation. In the case of SIRT1-mediated HIF-2α inactivation, contradictory findings have been observed [83–85], which might be explained by a cell type-dependent regulation [86]. Moreover, enhanced ROS levels were found to stabilize HIF-1 and HIF-2 by reducing the posttranscriptional hydroxylation of the HIF subunits most likely via inhibition of post-translationally hydroxylated by prolyl hydroxylases [87, 88].

Taken together, it is clearly reported that mitochondrial ROS accumulation correlates with HIF-α stabilization during hypoxia, while exogenous ROS enhance HIF-1 protein levels under normoxia [89], demonstrating the close relationship between the hypoxia- and redox-sensitive pathways.

### Evidence of compromised antioxidant defense in OA and OP patients

**Fig. 1:**
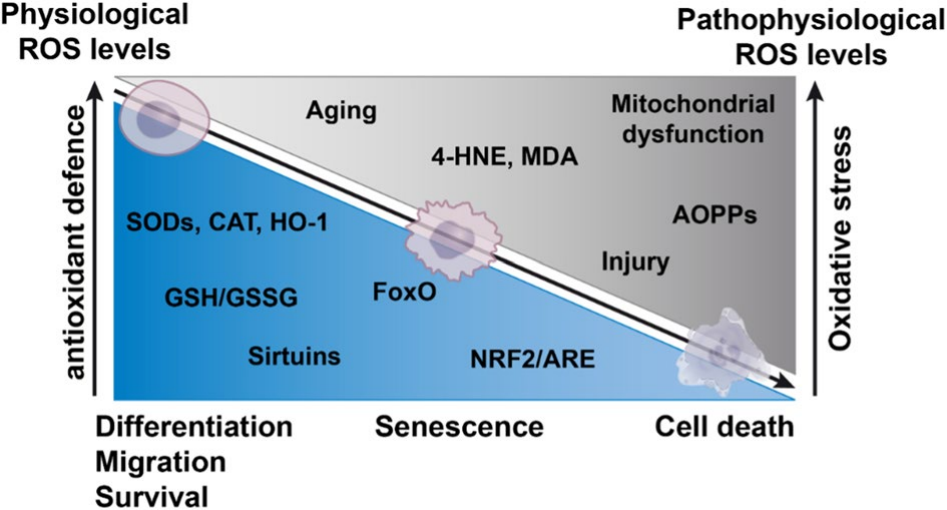
Overview of the redox balance in cell fate decision. The decrease of cellular antioxidant defense mechanisms (blue area) results in an increase of oxidative stress, driven and characterized by various factors (gray area). Alterations in the redox balance differentially effect cellular behavior and fate, ranging from physiological to pathophysiological processes

It is understood that enhanced ROS production and subsequent oxidative stress under pathophysiologic conditions mainly results from mitochondrial dysfunction, an upregulation of ROS-producing enzymes, and coincident impairment of the antioxidant defense system [90, 91]. The Nrf2 promoter consists of CpG islands, which are thought to be epigenetically modified upon aging, thus blocking the transcription of Nrf2 [92]. Accordingly, enhanced Nrf2 promoter hypermethylation was reported in both OP patients and ovariectomized mice [93]. In agreement with these findings, decreased Nrf2 protein levels were observed in cartilage of aged or OA donors and OA chondrocytes [94, 95]. This dysregulation of Nrf2 inevitably promoted mitochondrial dysfunction and thus enhanced ROS production [95–97]. The coincident reduction in cellular antioxidant expression results in a reduced ability to counter oxidative stress and thus enhanced cellular damage—a vicious cycle. An overview of the redox balance in cell fate decision, including proteins of the cellular antioxidant defense and ROS-associated markers, is given in Fig. 1.

Regarding cellular antioxidant enzymes, a significant correlation between decreasing GSH/GSSG and bone mineral destiny (BMD) was reported in postmenopausal women [98]. Accordingly, GSH/GSSG ratios were significantly lower in chondrocytes isolated from elderly donors as compared to cell derived from young donors [99]. This change in the GSH/GSSG ratio might result from a reduction in NADPH, the co-factor of glutathione reductase, the synthesis of which might be dysregulated in aging and senescence, as recently reviewed [100, 101]. In addition to the decline in active GSH, significantly lower SOD and CAT levels were found in respective tissue samples of OA and OP patients [93, 102–104]. Additionally, enhanced post-translational lysine acetylation and thus SOD2 inactivation was reported in human OA cartilage and in aged rats [105, 106]. Fu et al. described that the addition of SIRT3, which was substantially decreased upon aging, induced deacetylation, and subsequent SOD2 activation [105]. Although there are reports about a positive correlation between age and SIRT1 protein levels, implying a cellular response towards enhanced cellular stress [107], animal studies revealed a simultaneous decline in the enzymatic activity of the NAD-dependent deacetylase, which might result from an age-related decrease in systemic NAD^+^ biosynthesis [108]. Indeed, CD38 expression and activity, one of the main NADases, was found to be increased upon aging and cause the age-associated NAD reduction, promoting decreased sirtuin activity and enhanced mitochondrial dysfunction [109].

Taken together, the decrease in the cellular antioxidant defense system results in enhanced oxidative stress with age. Accordingly, enhanced levels of malondialdehyde (MDA) and 4-hydroxynonenal (4-HNE), the major aldehydic products and thus most commonly used biomarkers of lipid peroxidation, were found in cartilage as well as synovial fluid or cells of OA patients [106, 110–112]. Moreover, mitochondrial and genomic DNA damage in human chondrocytes was associated with age and OA [113, 114]. In postmenopausal women, a positive correlation between urinary 8-hydroxy 2′-deoxyguanosine (8-OHdG), a marker for DNA damage, and serum receptor activator of NFκB (RANK) ligand (RANKL) was found [115]. Furthermore, plasma NO and serum MDA levels were significantly higher in women suffering from OP as compared to the control group [116]. In agreement with this, ROS-related protein damage, which was determined by plasma advanced oxidation protein products (AOPPs) levels, was significantly elevated in postmenopausal OP women. These AOPPs levels were positively correlated with markers of bone turnover, bone-specific alkaline phosphatase, and tartrate-resistant acid phosphatase 5b and negatively correlated with lumbar BMD of the overall cohort [117].

## ROS-mediated cell fate decision in OA

OA is a multifactorial disease, mainly associated with age, sex, and obesity, but can also result from other risk factors, such as genetic predisposition and injuries of joint-related tissues. In contrast to idiopathic OA, which cannot be assigned to a certain inducer, posttraumatic OA (PTOA) is considered to be a result of preceding joint injuries. While PTOA accounts for only approximately 12% of all symptomatic OA cases, it has been presumed that between 20 and 78% of cases of ankle OA are posttraumatic [118, 119].

The pathomechanisms of idiopathic OA and PTOA are considered to be largely similar, comprising cell death, low-grade inflammation, oxidative stress, and consequent cellular dysfunction. The last is very complex and includes ROS- and/or cytokine-triggered chondrocyte phenotypical alteration, such as hypertrophy and senescence. Driven by the pathophysiologic conditions, hypertrophic and senescent cells exhibit a dysfunctional behavior characterized by excessive production of pro-inflammatory mediators and matrix-degradative enzymes, for example, matrix metalloproteinases (MMPs) and a desintegrin metalloproteinases with thrombospontin motive (ADAMTS), driving ongoing cartilage degeneration—the major hallmark of OA [120]. While chondrocyte hypertrophy is primarily considered as a physiologic mechanism associated to endochondral ossification in the growth plate during skeletal development or fracture healing, this so-called terminal differentiation of chondrocytes can also be observed in OA cartilage [121]. Although the underlying mechanisms promoting the phenotypical alteration from a mature articular chondrocyte towards the hypertrophic phenotype has not been completely understood, there is common agreement that this process is triggered by pathophysiologic conditions, including oxidative stress [122], and that accumulation of hypertrophic chondrocytes in cartilage tissue contributes to OA pathogenesis. According to their role during endochondral ossification, hypertrophic chondrocytes in OA cartilage not only express high levels of collagen type 10, but also catabolic (i.e., ADAMTS5 and MMP-13), pro-inflammatory (i.e., CXCL1 and IL-8), and ossification-related markers (i.e., RUNX2 and alkaline phosphatase) [123]. Regarding their secretome, hypertrophic chondrocytes share various markers with senescent chondrocytes [120], which will be described in the section “Role of ROS in SIPS” in more detail.

It should be noted that ROS-mediated changes in cellular function are not limited to chondrocytes. As a whole-joint-disease, several joint-related tissues and their respective cell types are affected by the pathophysiologic environment and oxidative stress. Accordingly, ROS accumulation modulates the behavior and fate of fibroblasts and macrophages in the synovial membrane or osteoblasts and osteoclasts in the subchondral bone. Similar to the phenotypical alterations in chondrocytes, synovial and bone-related cells develop a pro-inflammatory and catabolic phenotype, thus contributing to OA progression as reviewed elsewhere [11].

Besides the ROS-mediated phenotypical alterations in articular chondrocytes, the stressful environment may also result in chondrocyte death, which is similarly associated with cartilage destruction. As the exclusive cartilage-building cell type expressing collagen type II and aggrecan, the most abundant structural macromolecules in hyaline cartilage, mature articular chondrocytes are essential to maintain tissue homeostasis. A decline in chondrocyte number is highly detrimental due to the high hypocellularity of cartilage, which consists of only 2–5% of cells. Moreover, cell death and subsequent release of intracellular components and membrane fragments may function as damage-associated molecular patterns (DAMPs), further fueling inflammatory processes [124]. Thus, particularly lytic cell death, such as ferroptosis, necroptosis, and pyroptosis, contributes to progressive cartilage degeneration, creating a vicious cycle.

Taken together, both, chondrocyte cell death and survival represent a critical aspect in OA progression. Indeed, surviving chondrocytes might cause even more harm than those undergoing regulated cell death. This largely depends on two aspects: (i) the form of regulated cell death—either “clean” or “dirty”—and (ii) the phenotype of the surviving chondrocyte—either functional or dysfunctional. In short, a damaged chondrocyte undergoing apoptosis might be less harmful than an apoptosis-resistant, senescent chondrocyte, releasing proinflammatory and catabolic mediators.

In the following paragraphs, we will focus on the decisive role of ROS in cell fate decision of chondrocytes under pathophysiologic conditions.

### ROS in regulated cell death

Regulated cell death can occur as a physiologic process in embryonal development and plays a pivotal role in tissue homeostasis maintenance. It might at first appear paradoxical that cell death maintains tissue integrity, but elimination of damaged and thus dysfunctional cells indeed prevents chronic inflammation and diseases [125]. Potential consequences of insufficient clearance of dysfunctional cells are elaborated in the next chapter focusing on senescence (see “Role of ROS in stress-induced premature senescence”).

Several modi of regulated cell death have been described in OA, comprising apoptosis, necroptosis, pyroptosis, and ferroptosis [124, 126–128]. Although each type of cell death is characterized by different effector proteins, all of them can be directly or indirectly induced by oxidative stress. In terms of ROS-associated regulated cell death, p53 is considered to be a crucial key mediator [129]. In contrast to apoptosis, which is largely considered to be a clean and rather anti-inflammatory mode of regulated cell death, there are several pro-inflammatory and thus detrimental forms of regulated cell death. The pro-inflammatory feature of ferroptosis, necroptosis, and pyroptosis results from cell membrane disruption and the consequent release of intracellular components, which act as DAMPs. These mediators can bind to so-called pattern recognition receptors, such as toll-like receptors 2 and 4, on synovial cells and chondrocytes, thus promoting a pro-inflammatory response and consequent production of matrix-degradative enzymes [120].

In contrast to necrosis, which is characterized by passive membrane rupture, the loss of cell integrity in ferroptosis, necroptosis, and pyroptosis is a controlled process. In the case of necroptosis and pyroptosis, membrane permeabilization is mediated by integration of pore-forming oligomers of mixed lineage kinase domain-like (MLKL) and gasdermins (GSDMD), respectively. This mechanism appears to be similar to Bax/Bak-mediated mitochondrial outer membrane permeabilization in apoptosis [130].

**Fig. 2:**
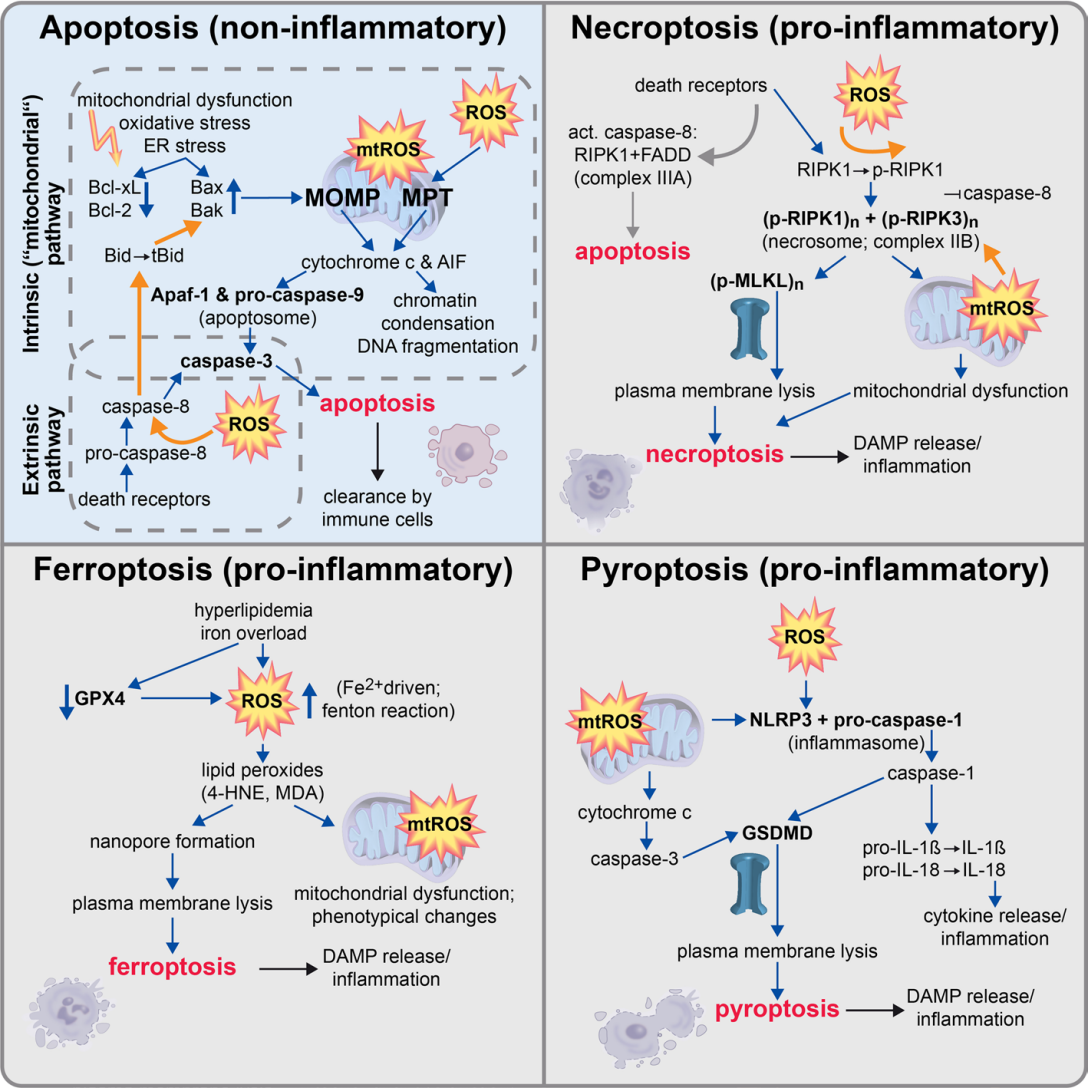
ROS involvement in regulated cell death. While apoptotic cell death (blue background) is considered as a largely silent and non-inflammatory form of cell death, other forms such as necroptosis, ferroptosis, and pyroptosis (gray background) are thought to promote a pro-inflammatory response. Regardless of the pathophysiologic consequences, ROS and mitochondrial dysfunction (mtROS) play a decisive role in the execution of all described forms of regulated cell death. *Apaf-1* apoptosis activating factor-1, *DAMP* damage-associated molecular patterns, *GPX4* glutathione peroxidase 4, *GSDMD* gasdermins, *MOMP* mitochondrial outer membrane permeabilization, *MPT* mitochondrial permeability transition, *(mt)ROS* mitochondrial) reactive oxygen species, *NLRP3* nucleotide-binding domain and leucine-rich repeat protein-3

In the following section, we will give a short overview of apoptotic, ferroptotic, necroptotic, and pyroptotic cell death, and particularly focus on the executive role of oxidative stress in this context. The involvement of ROS in the different modi of cell death is outlined in Fig. 2.

#### Apoptosis

In general, the plasma membrane of apoptotic cells remains intact during the self-elimination process, preventing the release of intracellular components, which might act as immunogenic DAMPs. Therefore, apoptosis has commonly been considered to be an “immunologically silent” form of cell death. However, recent studies refute this theory, because apoptotic cells have indeed been described to release DAMPs under certain circumstances [131]. Currently, apoptotic cells are regarded to be metabolically active and highly communicative cells, which exhibit a distinct secretome, including nucleosome components and pro-regenerative apoptotic extracellular vesicles, as well as anti-inflammatory and immunomodulatory metabolites [132–134]. These new findings imply that apoptotic cells might be involved in the modulation of posttraumatic and pathophysiologic processes, thus being more than just damaged cells waiting for phagocytic clearance [133].

Mechanistically, two main pathways of apoptosis can be distinguished—the intrinsic and extrinsic pathways—which can both be activated by ROS as previously reviewed [135]. In the case of intrinsic apoptosis, which is mainly associated with mitochondrial dysfunction or ER stress, ROS can activate the cell cycle regulators p53 and JNK, which induce pro-apoptotic Bcl-2 family proteins such as Bak and Bax—antagonists to anti-apoptotic proteins Bcl-xL and Bcl-2 in the outer mitochondrial membrane (OMM)—eventually causing mitochondrial outer membrane permeabilization and subsequent apoptosis [136, 137]. At low stress levels and potentially reversible cellular damage, p53 induces a cell cycle arrest allowing DNA repair. In this case, cell fate is thought to be mainly influenced by the p53 level, including a complex regulation mechanism of posttranscriptional modification and thus activation of the cell cycle regulator, occurring in pulses [138]. However, p53-induced cell cycle arrest might also lead to senescence.

Moreover, ROS accumulation results in oxidation of cardiolipin, the signature phospholipid in the inner mitochondrial membrane, as well as mitochondrial depolarization and subsequent opening of Bax/Bak channels in the OMM, resulting in mitochondrial outer membrane permeabilization. Taken together, these processes cause the release of pro-apoptotic mediators, such as cytochrome c and apoptosis-inducing factor (AIF). AIF is translocated to the nucleus, where the protein causes chromatin condensation and DNA fragmentation, thus initiating caspase-independent apoptosis. By contrast, cytochrome c interacts with apoptosis activating factor-1 and procaspase-9, forming the apoptosome. Subsequent autoactivation of caspase-9 initiates the caspase cascade by activation of the downstream executioner caspases-3, -6, and -7 [139]. Release of pro-apoptotic cytochrome c and AIF can also occur upon ROS-mediated mitochondrial permeability transition, leading to osmotic swelling and OMM rupture [140].

In addition to intrinsic apoptosis, ROS were found to be involved in extrinsic apoptosis mediated via transmembrane death receptors, comprising Fas, tumor necrosis factor (TNF)-related apoptosis-inducing ligand receptor 1/2, and TNF receptor 1 (TNFR1). Indeed, the extrinsic pathway, which mediates caspase-8 activation and caspase-3 and -7 engagement, similarly to the intrinsic pathway, was found to be rather inefficient. Therefore, a crosstalk between the extrinsic and intrinsic (“mitochondrial”) pathways exists, which amplifies extrinsic death signals by additional ROS production [135].

In OA, ROS-induced apoptosis is considered to be the predominant form of chondrocyte death, in particular, after joint injuries. There is strong evidence that posttraumatic ROS accumulation might primarily result from mitochondrial dysfunction [141]. Accordingly, mitochondrial stabilization by the mitoprotective drug SS-31 significantly ameliorated chondrocyte apoptosis by attenuating strain-dependent mitochondrial ROS (mtROS) production [142, 143]. Moreover, Nrf2/ARE-pathway induction and consequent expression of antioxidant target genes, for example, HO-1 and SOD2, enhanced the anti-apoptotic response in chondrocytes [144]. Similar anti-apoptotic effects in chondrocytes were reported for the deacetylase SIRT1, which was ascribed to different potential mechanisms: First, SIRT1 promotes p53 deacetylation, thus serving as a negative regulator to apoptosis-associated Bcl-2 decline and Bax increase [145]. Second, particular cell protective effects of SIRT1 were reported to result from autophagy or mitophagy activation [146].

#### Ferroptosis

Ferroptosis is a relatively newly discovered form of regulated cell death, which was initially described as iron-dependent. To date, the exact underlying mechanisms of this ROS-dependent mode of cell death have not been completely elucidated. In addition to oxidation of labile iron, GPX4 inactivation and subsequent lipid peroxidation have been identified as the main hallmarks of ferroptosis [147]. On a biochemical level, GSH depletion and impaired GPX4 activity as well as Fe^2+^-driven ROS production (Fenton reaction) result in oxidative stress and lipid peroxide accumulation, initiating cell death [148]. Nanopore formation and consequent Ca^2+^ influx eventually results in membrane rupture and the release of pro-inflammatory cell components [149].

Whether iron is essential in lipid peroxide production and subsequent initiation of ferroptosis remains to be clarified. However, there are a number of mainly GPX4-targeted drugs and compounds, which are known to induce ferroptotic cell death, including GPX4-degrading or -inhibiting substances as well as inhibitors of the cystine-glutamate antiporter system, pronouncing the decisive role of the antioxidant system in ferroptosis [150]. In comparison to other common forms of cell death, such as apoptosis or necroptosis, ferroptotic cells have a unique mitochondrial phenotype, characterized by shrinkage and increased membrane density of this organelle, as well as crista reduction or loss and OMM rupture [147, 151]. Moreover, it is assumed that changes in the mitochondrial metabolism and thus excessive mtROS production might promote ferroptosis [148].

Both hallmarks of ferroptosis, iron dyshomeostasis and enhanced lipid peroxidation, are strongly associated with OA, as previously reviewed elsewhere [152]. In brief, increased of Fe^2+^, Fe^3+^, and total iron concentrations, as well as the main reactive products of lipid hydroperoxides, 4-HNE and MDA, have been reported in cartilage, synovial tissue and synovial fluid of OA patients. At the same time, reduction in GPX and GSH levels or the GSH/GSSG ratio was observed in plasma, cartilage tissue, and synovial fluid, respectively.

The crucial role of ferroptotic cell death in OA progression was recently confirmed in rodent PTOA models [153–155]. Interestingly, antioxidant treatment with natural phenol theaflavin-3,3′-digallate protected human chondrocytes from erastin-induced ferroptosis by promoting the Nrf2-pathway and thus increasing GPX4 and HO-1 expression. Accordingly, theaflavin-3,3′-digallate treatment prevented injury-induced chondrocyte ferroptosis and OA progression in vivo [155]. Similarly, anti-ferroptotic and thus chondroprotective effects of the iron-chelating agent deferoxamine were found to be mediated by enhanced Nrf2 signaling and subsequent increase of the antioxidant defense system in chondrocytes [154].

#### Necroptosis

In the case of necroptosis, the initial steps are similar to those of extrinsic apoptosis. In brief, TNFR1 activation by TNF results in TNFR1 complex I formation, which includes the receptor-interacting serine/threonine-protein kinase 1 (RIPK1)—a key regulator of diverse cellular processes ranging from inflammation and cell survival to apoptosis and necroptosis. The mode of regulated cell death is determined by the presence (apoptosis) or absence (necroptosis) of active caspase-8 [156].

Following TNFR1 complex I assembly, caspase-8 inhibition activates the necroptotic pathway by the interaction of RIPK1 and RIPK3, which form the so-called necrosome (complex IIB) [157]. Subsequently, the MLKL is recruited and phosphorylated, initiating MLKL oligomerization. Integration of the pore-like oligomers into the cell membrane results in sodium and calcium influx and potassium efflux, eventually leading to its disruption [158, 159]. Indeed, mtROS were observed to induce RIPK1 autophosphorylation and consequent activation of the apoptotic and necroptotic pathways [160]. Moreover, there is evidence that RIPK1, RIPK3, and/or MLKL are translocated to the mitochondrial membrane, initiating mtROS production and subsequent execution of necroptotic cell death, as reviewed elsewhere [161]. It is assumed that the necrosome activates further target proteins, such as the mitochondrial phosphatase PGAM5, which interacts with RIPK3, thus inducing mitochondrial fragmentation in a dynamin-related protein 1 manner [162]. Furthermore, RIPK3 was found to activate the rate-limiting enzyme pyruvate dehydrogenase complex, which connects glycolysis to aerobic respiration. This activation results in enhanced aerobic respiration and higher mtROS production [163]. Interestingly, MLKL was required for RIPK3-mediated phosphorylation of PGAM5 and pyruvate dehydrogenase complex, implying that RIPK3 translocation to the mitochondrial target proteins might depend on necrosome formation [162, 163].

In agreement with the aforementioned reports, addition of ROS-scavengers, for example, butylated hydroxyanisole, or electron transfer chain inhibitors, such as amytal (amobarbital), were found to suppress TNF-induced necroptosis [160, 164, 165]. In addition, adenoviral overexpression of mitochondrial SOD2 was observed to protect endothelial cells from NO-mediated accumulation of superoxide in mitochondria and consequent necroptosis [166]. The pro-necroptotic role of ROS was also confirmed in an ex vivo human cartilage trauma model, in which antioxidative therapy using N-acetyl cysteine efficiently protected chondrocytes from injury—and TNF/cycloheximide-induced apoptosis as well as necroptosis [124, 128]. Moreover, it could be demonstrated that addition of the pan-caspase inhibitor zVAD after cartilage trauma or during TNF/cycloheximide stimulation resulted in a shift from apoptosis to necroptosis in chondrocytes. This increase of necroptotic cells was associated with enhanced release of intracellular DAMPs as well as elevated NO and prostaglandin E2 production [124], which confirms the pro-inflammatory consequences of necroptosis.

#### Pyroptosis

The nucleotide-binding domain and leucine-rich repeat protein-3 (NLRP3) inflammasome, also referred to as NLR family pyrin domain containing 3, is a stress sensor and key mediator of inflammation—associated with many inflammation-related diseases [167]. In addition to NLRP3, the NLRP3 inflammasome consists of a caspase recruitment domain (CARD)-containing adaptor protein termed apoptosis-associated speck-like protein containing a CARD and pro-caspase-1. In the canonical pathway, NLRP3 activation induces autoproteolysis of pro-caspase-1 into the effector caspase-1. Active caspase-1 further leads to the cleavage of pro-interleukin-1β (pro-IL-1β), pro-IL-18, and GSDMD, initiating oligo-GSDMD pore formation and subsequent cytokine release and pyroptosis [127, 168].

There is evidence that oxidative stress represents a major trigger of inflammasome activation and consequent pyroptotic cell death [169]. In this context, elimination of dysfunctional mitochondria and thus reduction of excessive mtROS production was found to have an attenuating effect on pyroptosis [170]. ROS-induced activation of the NLRP3 inflammasome is thought to be mediated by the thioredoxin interacting protein detaching from thioredoxin under oxidative stress [171]. Furthermore, ROS-related pyroptosis might also be induced by activation of redox-sensitive pathways, including ERK and p38 MAPK as well as NFκB [172]. By contrast, the Nrf2/HO-1 signaling pathway was found to attenuate the expression of NLRP3 as well as of caspase-1 and IL-1 β in various cell types [173, 174]. In line with this, Nrf2/HO-1 pathway inhibition was hypothesized to promote pyroptosis in OA [175], while enhanced Nrf2 signaling by the addition of the antihistamine loratadine or the flavonoid licochalcone A was described to alleviate NLRP3-related pyroptosis as well as consequent inflammation and OA progression in human chondrocytes and in a murine injury-induced OA model, respectively [176]. Moreover, pioglitazone-mediated peroxisome proliferator-activated receptor γ (PPAR-γ) activation alleviated lipopolysaccharide/ATP-induced pyroptosis and arthritis by promoting both, the Nrf2- and PPAR-γ coactivator-1 /Δψm signaling pathways [177]. The latter has been described to support mitochondrial function under oxidative stress and increase mitochondrial biogenesis as well as mitophagy [178, 179]. Further evidence of the decisive role of ROS in pyroptotic cell death was provided by Lui et al., who observed that ubiquitin-specific protease 7 overexpression results in NOX4-dependent ROS production and chondrocyte pyroptosis. Ubiquitin-specific protease inhibition, by contrast, prevented NLPR3 inflammasome formation, IL-1β and IL-18 production, pyroptosis, and monosodium iodoacetate-induced OA in mice [180].

### Role of ROS in SIPS

Cellular senescence is described as an irreversible state, characterized by alterations in the cell-cycle regulation leading to permanent cell cycle arrest. In general, cellular senescence is not detrimental per se. It plays an important role in embryonic development, wound healing, and tumor suppression [181]. However, chronic senescence has been described as a pathophysiologic process in age-related and degenerative diseases [182]. In contrast to replicative senescence, which is associated with a limitation in cell division through telomere shortening (Hayflick limit) [183, 184], stress of a physical or chemical nature and consequent DNA damage and/or oxidative stress, has been considered to be a major driver of so-called SIPS. Both, replicative senescence and SIPS share similar characteristics, including changes in cell morphology and enhanced senescence-associated β-galactosidase (SA-β-Gal) activity [185]. About two decades ago, Price et al. were the first who described that human chondrocytes located in close proximity to OA lesions were stained positively for SA-β-Gal and had a shortened telomere length [186]. Furthermore, it was demonstrated that senescent chondrocytes accumulate in human articular cartilage with increasing age or after injury, which was hypothesized to be driven by oxidative stress as one of the main triggers [187, 188].

Another hallmark of senescence—and maybe the most detrimental one in regards to tissue homeostasis—is the senescence-associated secretory phenotype (SASP). Transient senescence and consequent SASP mediator release are thought to promote tissue repair and regeneration, whereas an enduring senescence can lead to chronic inflammation [189, 190]. Under physiologic conditions, SASP attracts immune cells which eliminate damaged and senescent cells. This mechanism can be impaired during aging because of immune system dysfunction [191]. Due to the inefficient removal of damaged cells in cartilage tissue, recent studies suggested that senescent chondrocytes substantially contribute to OA progression most likely due to excessive secretion of SASP factors [120, 192]. ROS and mitochondrial dysfunction play an important role in chondrocyte production of SASP components, which is mainly driven by the NFκB signaling pathway [193, 194]. The SASP secretome of senescent chondrocytes contains proinflammatory cytokines and chemokines (e.g., IL-6, IL-8, and CXCL1) as well as proteases (e.g., MMPs and ADAMTS), creating a degenerative microenvironment [120]. Moreover, SASP factors contribute to the spreading of senescence [195, 196], therefore, leading to an accumulation of dysfunctional, senescent cells. Accordingly, intraarticular injection of senescence cells resulted in enhanced pain, impaired mobility, and OA-associated radiographic and histological alterations of the joint in healthy mice [192].

In regards to oxidative stress, increased ROS levels induce directly (via MKK3/6-p38 pathway) or indirectly (via DNA-damage response and ATM/ATR pathway) the transcription of p16 [also known as cyclin-dependent kinase inhibitor 2A (CDKN2A)], p53 (encoded by *TP53*), and p21 (encoded by *CDKN1A*) [193, 197]. These proteins are involved in cell cycle regulation and promote a non-proliferative phenotype upon activation. The transcription factors p16 and p21 are both cyclin-dependent kinase inhibitors, which bind to CDK4/6 and CDK2, respectively, thus controlling retinoblastoma protein activity and preventing entry into the S phase of the cell cycle [182]. Furthermore, the p53-p21 pathway appears to play a crucial role in cell fate decision by inhibiting or inducing apoptosis in a damage-dependent manner. Human diploid fibroblasts treated with low, sublethal H_2_O_2_ doses, were found to undergo a senescent-like growth arrest. Higher H_2_O_2_ concentrations, in turn, induced apoptosis and led to increased p53 levels compared to growth-arrested cells [198]. Induction of DNA damage and apoptosis via caspase 3, caspase 8, and caspase 9 by high H_2_O_2_ levels was also observed in human bronchial epithelium cells [199]. In agreement with this, exposition to low H_2_O_2_ or doxorubicin doses represent frequently used models to study SIPS in human chondrocytes, while high doses resulted in ROS-mediated apoptosis [200, 201]. In addition to ROS and p53 levels, another cell cycle regulator might play a substantial role in cell fate decision. Yosef et al. showed that p21 knockdown leads to a decreased cell survival of senescent cells, implying a shift from senescence towards apoptosis in the absence of p21 [202]. Overall, it has been proposed that the p53-p21 pathway is essential in initiating senescence [203], whereas p16 is assumed to maintain the senescent phenotype [204, 205]. Numerous studies showed that p21 decreases in senescent cells with time, whereas the p16 level remains elevated [206, 207]. Additionally, it was demonstrated that p53-induced senescence could be reversed by p53 inactivation in cells with low p16 activity. However, when p16 was present at high levels, p53 inactivation was insufficient to restore cell replication [208]. Although p16 expression was observed to correlate with several SASP factors in human chondrocytes, neither SASP expression nor OA severity was attenuated by somatic p16 inactivation in chondrocytes of adult mice [209]. It was concluded that p16 is a biomarker for dysfunctional cells, but the driver of OA is probably the extensive SASP mediators release by senescent chondrocytes rather than impaired replication potential. This assumption is supported by Coppé et al., who provided evidence that p16 is not required for SASP production and suggested the secretory phenotype to be a damage response which might occur independently of the cell cycle arrest [210]. Overall, it remains to be clarified whether the loss of replicative function of mature and thus postmitotic chondrocytes is relevant in OA progression.

ROS-mediated damage of mtDNA and lipid peroxidation is known to result in mitochondrial dysfunction and thus excessive ROS production due to inefficient electron transfer. In this way, mitochondrial dysfunction promotes senescence and vice versa [211, 212]. One mechanism to restore the function of damaged mitochondria is the fusion with a healthy one to replace damaged lipids. However, in senescent cells, the mitochondrial dynamics are impaired, frequently leading to elongated, enlarged, and hyperfused mitochondria [213, 214]. The large size of mitochondria in senescent cells compromises the removal of dysfunctional mitochondria by means of mitophagy. Moreover, it has been demonstrated that p53 is not only involved in senescence induction, but is also able to inhibit mitophagy-mediated mitochondrial degradation through interaction with Parkin [215, 216]. Additionally, the expression of PINK1, which is involved in mitophagy and mitochondrial fission, appears to be diminished in senescent cells [211]. In a murine OA model, oral application of the mitophagy-inducing drug Urolithin A was found to prevent cartilage degradation and reduce synovial inflammation as well as the expression of the SASP factors p16 and p21 in senescent human chondrocytes [217, 218]. Together, these studies suggest that impaired mitochondrial dysfunction and disturbed mitophagy both represent a substantial therapeutic target against chondrosenescence.

Furthermore, recent studies demonstrated that Sirt1 and Sirt6 are downregulated in senescent chondrocytes. While Sirt1 suppresses the expression of SASP factors, Sirt6 presumably promotes DNA repair [219]. Accordingly, increased Sirt1 and Sirt6 expression attenuates OA progression by diminishing chondrocyte senescence [220–222]. Together, these reports suggest that an upstream targeting by means of antioxidants or mitophagy-inducing drugs might be promising to prevent ROS-induced premature chondrocyte senescence. However, most pharmacological strategies against chondrosenescence focus on the stabilization of the chondrocyte phenotype by means of senomorphics or selective elimination of dysfunctional cells by senolytics [223]. The latter approach has currently gained considerable attention. Indeed, selective removal of senescent chondrocytes by senolytic-induced apoptosis attenuated post-traumatic OA progression and enhanced cartilage regeneration in mice [188]. These data not only underline that senescence could be a promising target for preventing or delaying OA, but also that the survival of damaged and thus dysfunctional cells might be more detrimental than regulated cell death.

**Fig. 3:**
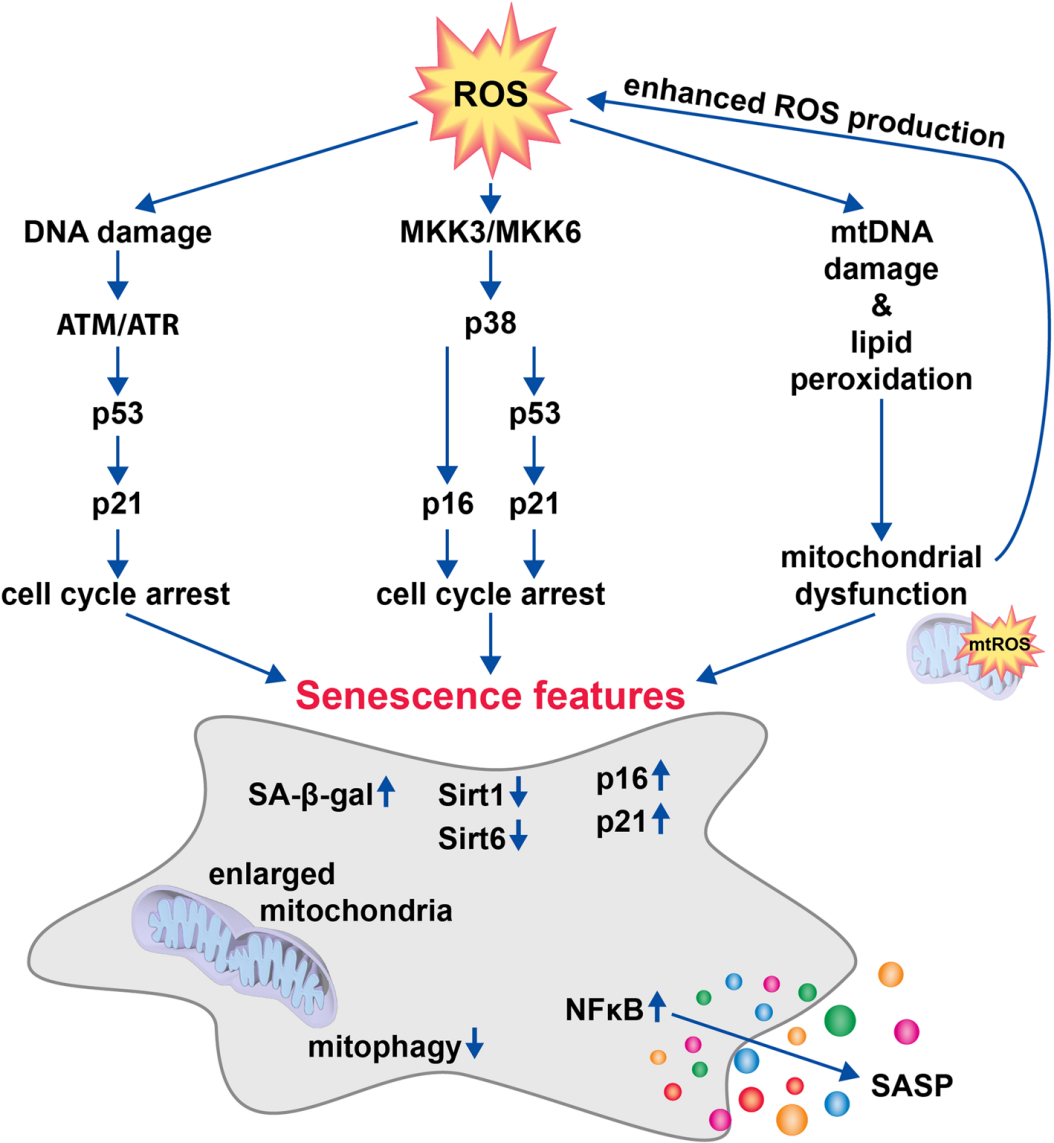
ROS as a driver of senescence. Enhanced ROS levels result in cellular damage and consequent cell cycle arrest mediated via p53, p21, and p16. Moreover, ROS damages mitochondrial DNA (mtDNA) and causes lipid peroxidation initiating mitochondrial dysfunction and thus lead to enhanced ROS generation. Other senescence features are the expression of senescence-associated β-galactosidase (SA-β-Gal), downregulation of Sirt1 and Sirt6, upregulation of p21 and p16, presence of enlarged mitochondria, inhibition of mitophagy, and secretion of senescence-associated secretory phenotype (SASP) factors mainly driven by the NFκB

The involvement of ROS in cellular senescence and the main hallmarks of senescent cells are outlined in Fig. 3.

## ROS-mediated cell fate decision in bone remodeling and fracture healing under physiologic conditions

During early life, the balance between bone formation by osteoblasts and bone resorption by osteoclasts is strictly regulated. Bone remodeling is locally mediated by growth factors, such as members of the transforming growth factor β superfamily, and cytokines, such as IL1, IL6, and TNF, as well as systemically by hormones and vitamins, including parathyroid hormone, estrogen, and vitamin D. Osteoclast precursors differentiate in response to the monocyte/macrophage colony-stimulating factor and RANKL, which are expressed by, for example, osteoblasts and stromal cells. Osteoprotegerin (OPG), similar to RANK, the receptor of RANKL, is a receptor of the TNF family and acts as a decoy receptor for RANKL, thereby inhibiting osteoclast differentiation and activity. The balance of RANKL/OPG is regulated by various osteotropic factors, including estrogen, parathyroid hormone, IL1, IL17, and TNF.

In addition to the above-mentioned mediators, ROS play a critical role in the regulation of physiologic bone remodeling and fracture repair, having striking effects on both osteoblast and osteoclast lineage activities, including replication of undifferentiated cells, cell recruitment, and function of differentiated cells [90, 224–227]. Under healthy conditions, ROS generated by osteoclasts activate and keep bone resorption in balance with osteoblast bone formation. Exogenous ROS, mainly H_2_O_2_ and superoxide, result in osteoclastogenesis and osteoclast activity by initiating RANK signaling in macrophages and, in turn, endogenous ROS generation in osteoclasts is stimulated by RANKL to enable bone resorption. Endogenous ROS production activates TNF receptor associated factor 6, NOX1, and the transcription factors NFκB and nuclear factor of activated T-cells [226]. This cell type increases the expression of Tartrate-resistant acid phosphatase 5b, a multifunctional enzyme, which promotes ROS generation and is significantly elevated in OP patients [228].

Bone fracture healing is a complex and dynamic process that involves the coordinated activity of various cell types and molecular mechanisms, resulting in an uneventful repair of the injured bone under physiologic conditions. The regeneration starts with an inflammatory phase, characterized by a high abundance of cells of the innate and adaptive immune systems, which remove cell debris and secrete various pro- and anti-inflammatory cytokines and mediators [229]. These factors recruit mesenchymal and vascular progenitor cells to the injury site, initiating the repair phase. This phase, which lasts for several weeks following the fracture, is characterized by the formation of a soft callus, which consists of fibrous tissue, cartilage, and woven bone. Mesenchymal stem cells (MSCs) differentiate into chondrocytes, which produce cartilage, or osteoblasts, which produce bone [230]. Additionally, blood vessels grow into the callus, providing oxygen and nutrients to support tissue growth. During endochondral ossification in the fracture callus, chondrocytes can directly undergo transdifferentiation to osteoblasts, thereby contributing to the overall bone formation. During the remodeling phase, which can last for several months or even years following the fracture, the callus is gradually replaced by mature bone tissue. Osteoblasts and osteoclasts collaborate to resorb the newly formed bone tissue and replace it with more structurally sound bone, respectively. Many factors that can affect the rate and quality of fracture healing include age, nutrition, and the presence of comorbidities such as diabetes or OP [231].

Oxidative stress plays a crucial, but multifaceted role during bone regeneration [232]. During the early stages of fracture healing, oxidative stress is involved in the inflammatory response. ROS are mainly generated by immunomodulatory cells [233] and are thought to contribute to the recruitment of further immune cells to the fracture site and to the induction of neovascularization [234]. It is known that low ROS levels stimulate angiogenesis and, therefore, blood flow in a vascular endothelial growth factor-dependent manner [234], which indicates an important role of endogenous ROS production in the early phase of fracture healing. During chondrocyte transdifferentiation to osteoblasts in the fracture callus, ROS are also important because they stimulate hypertrophy in chondrocytes and induce mineralization via the ERK and p38 MAPK pathways [122]. Therefore, ROS are crucial for cell fate decision during endochondral ossification. Additionally, ROS can activate signaling pathways that promote the proliferation and differentiation of bone-forming cells [235]. Oxidative stress is critically involved in mitochondrial fragmentation, which is associated with osteoblast differentiation via the ERK1/2 pathway. By contrast, excessive ROS production during this stage can also contribute to tissue damage and thus impair fracture healing. In particular, during the later stages of fracture healing, oxidative stress can have detrimental effects. Excessive ROS production is known to disturb bone-forming cell function, influence cell fate decision in mesenchymal stem cells (MSCs) towards the adipogenic lineage, and impair the production of extracellular matrix proteins [236], as described in more detail below. Moreover, excessive ROS production increases osteoclast differentiation from macrophages by RANK signaling as described above, supporting enhanced bone resorption during fracture callus development [237]. Additionally, excessive oxidative stress can also impair angiogenesis, which is critical for the delivery of nutrients and oxygen to the fracture site [232, 238]. This can lead to delayed healing and an increased risk of non-union.

One aspect of the fracture healing process that has received increasing attention in recent years is the role of cellular senescence. As described above, oxidative stress signaling is critically involved in stress-induced premature cell senescence. In the context of fracture healing, senescence per se has been shown to play a role in both the early and late stages of this process [239, 240]. During the inflammatory phase of fracture healing, senescent cells are thought to contribute to the clearance of damaged tissue by cytokine secretion and promote the differentiation of mesenchymal stem cells into osteoblasts [241]. However, senescence can also have adverse effects on fracture healing, particularly when senescent cells accumulate in the later fracture callus [240, 242]. Saul et al. demonstrated that the highest accumulation of senescence marker during fracture healing occurs in the mesenchymal cell population. These cells produce extracellular matrix proteins that are less functional than those produced by non-senescent cells, which may reduce the quality of the new bone tissue. Furthermore, senescence can impair the ability of cells to respond to mechanical stimuli by inducing changes in the cell’s cytoskeleton [243]. Because mechanotransduction is critical for efficient fracture healing, ROS-induced senescence might hamper the regeneration process. The expression of SASP proteins like IL-6 or TNF by senescent cells might also impair fracture healing. Indeed, treating mice with senolytics during fracture healing reduced the expression of SASP markers and significantly accelerated bone regeneration [240, 244].

## ROS-mediated cell fate decision in OP

OP is globally the most common metabolic- and inflammation-associated bone disorder [245, 246]. It is characterized by reduced bone mass and a degenerated bone microarchitecture. Compromised bone strength predisposes OP patients to a higher susceptibility to fracture. The bones become weak and brittle, thus, patients suffer from fragility fractures, which may arise even from a low-impact fall. Such OP fragility fractures predominantly occur at the spine and the hip, however, other anatomical structures, including the proximal humerus and the distal forearm, can also be affected [247]. The average lifetime risk (%) of a 50-year-old woman to suffer a major OP fracture has been calculated at approximately 50% and in men at 22% [248, 249]. There are two types of OP, which are generally distinguished: Primary OP that can generally affect individuals of both sexes and all ages, but frequently appears after menopause in women through estrogen decline (type I OP) or is associated with age in women and men (type II OP, also termed as senile OP). Secondary OP results from prolonged therapeutic drug treatments, medical disorders, or a decrease in physical activity [245, 246].

Overall, oxidative stress is known to play a substantial role in the pathogenesis of OP. In the case of OP type I, for example, estrogen deficiency was demonstrated to inhibit mitochondrial β-oxidation of fatty acids, thus elevating ROS generation in mitochondria and peroxisomes [250]. In general, ROS accumulation has been associated with osteoblast and osteocyte apoptosis, as well as an impairment of both, mineralization and osteogenesis in bone [90, 251, 252]. Moreover, redox imbalance mediated by excessive ROS production causes elevated osteoclast differentiation from macrophages, supporting enhanced bone loss and thus OP development [237]. In the following paragraphs we will focus on two major pathophysiologic aspects in OP bone; (i) ROS-mediated disturbance of immune and bone cell fate regulation, and (ii) mitochondrial dysfunction resulting from compromised mitophagy.

### Role of ROS in immune cell regulation

The term “immunoporosis” emphasizes the significant role of both, innate immune cells and cells of the adaptive immune system in the pathogenesis of OP [253]. Cells from the innate immune system share the developmental niche with bone cells, indicating the close relationship between the immune system and the skeletal tissue. Myeloid lineage cells, such as macrophages, monocytes, dendritic cells, neutrophils, and mast cells as well as cells of the lymphoid lineage, such as natural killer and innate lymphoid-like cells, are involved in OP development by secreting various pro-inflammatory factors [253–255]. In addition to cytokines, including IL-6, IL-1β, TNF-α, and interferon (IFN) γ, immune cells produce high levels of ROS, thus activating osteoclastogenic bone resorption. Overall, immune cell-derived ROS and pro-inflammatory cytokines interact directly or indirectly with bone cells and contribute to an inflammatory state, driving the pathogenesis of OP.

Macrophages are the predominant cells in mediating the inflammatory response upon traumatic injuries and aging [226, 256]. The bone microenvironment can influence the metabolic state and the interaction of osteoclasts with macrophages. Depending on the local conditions, bone marrow macrophages can be polarized into M1 (pro-inflammatory) or M2 (anti-inflammatory) macrophages. Inflammatory stimuli, including ROS generated from damaged or necrotic tissue, cause a shift in macrophage polarization from the M2 to the M1 phenotype, the latter being associated with an elevation in cytokines and thus further immune cell recruitment. Moreover, M1 macrophages themselves generate high levels of ROS that reinforce osteoclastogenesis and bone resorption [226]. Besides macrophages, mast cells can be found in various connective tissues, including bone tissue. Under inflammatory conditions, mast cells become activated by ROS and are able to promote osteoclastogenesis by producing pro-inflammatory mediators, such as histamine, enzymes, and various cytokines released from their secretory lysosomes, also called granules [257, 258]. As mentioned above, age-related inflammation and oxidative stress promote cellular senescence. The SASP of senescent cells augments both, myelopoiesis and immune cell recruitment, including myeloid-derived suppressor cells, regulatory monocytes, and macrophages (regulatory macrophages/inactivated M2c macrophages) into the aging tissue. This inflammatory condition mediated by senescent cells leads to a compensatory immunosuppression, which restrains the low-grade inflammation in the aged tissue [259, 260]. Accordingly, immunosuppression hampers the elimination of senescent cells, which results in a negative feedback-loop on immunosuppressive cells, enhancing their release of inflammatory cytokines and ROS, thereby modulating other immune cells, inhibiting the immune response, and impairing tissue homeostasis [259, 260].

Accompanying the low-grade inflammation in aging tissues, including bone, the function of the immune system decreases continuously—a ROS-driven process that is termed immunosenescence. One hallmark of immunosenescence in aging is the decrease of cells of the adaptive immune system. Lymphocytes are the representatives of the adaptive immune system, consisting predominantly of T and B lymphocytes. A decline in naïve CD4^+^ and CD8^+^ T-cells, and simultaneous induction of regulatory T cells by myeloid-derived suppressor cells has been demonstrated in immunosenescence [260, 261]. Considering the high expression of Nrf2 and thus ARE-regulated antioxidant enzymes in myeloid-derived suppressor cells, it is unsurprising that this cell type exhibit an enhanced resistance to ROS. Oxidative stress is thought to promote T cell activation and their differentiation in so-called Th17 cells. These inflammatory T cells stimulate osteoclastogenesis in a direct or an indirect manner and also inhibit osteoblastogenesis by producing cytokines, such as IL-6, IL-17, and TNFα [262]. Recent studies suggested that B cells are involved in the regulation of the RANKL/OPG axis, and RANKL generation by B cells promotes the bone mass loss caused by estrogen deficiency [263].

In postmenopausal OP (OP type I), estrogen deficiency is strongly associated with immunosenescence that results in oxidative stress and bone loss in women [255, 262]. Notably, it has been demonstrated that the decline in estrogen levels and consequent immunosenescence is characterized by reduced IFNγ, decreased T lymphocyte production and proliferation, and a reduction of antioxidant enzymes, including SODs and CAT activities in peripheral blood mononuclear cells [264]. Regulatory B cells, a subset of B cells, are, under physiologic conditions, able to suppress the action of cytokines and osteoclast stimulators, such as IL-1 and TNFα, as well as T cells, like Th17 cells that are involved in inflammatory bone loss. Indeed, the impairment of regulatory B cells together with the inability to release IL-10 might be a contributory factor towards the establishment of pro-inflammatory conditions leading to bone loss in postmenopausal OP [265]. Taken together, immunosenescence is not only a result of oxidative stress, but also a potential cause of excessive ROS generation in OP bone.

### Role of ROS in bone cell fate regulation

Bone marrow-derived MSCs that give rise to the osteoblast lineage, together with osteocytes, are long-lived cells in the skeletal tissue, and, therefore, more predisposed to molecular changes during aging [300]. Accordingly, impaired MSC activity and function, including compromised osteogenic differentiation and commitment, appear to be responsible for the reduced number of osteoblasts in aging bone. Therefore, enhanced ROS levels caused by estrogen deficiency can influence the cell fate of both osteoprogenitor cells and mature osteoblasts by altering their biosynthetic activity and inducing senescence or apoptosis in OP [251]. Moreover, studies have shown that ROS promote the commitment of mesenchymal progenitor cells to the adipocyte lineage at the expense of osteogenic differentiation. Indeed, ROS-mediated lipid oxidation causes PPARγ interaction with β-catenin, inducing degradation of the latter, and thereby impairing Wnt/β-catenin signaling-related bone formation. In addition, the decrease in wnt/β-catenin signaling is suggested to be responsible for the increase of bone marrow adipose tissue and bone loss in skeletal aging [266].

Osteocytes are terminally differentiated osteoblasts and are embedded in lacunae of the mineralized bone matrix. They are the most abundant cells (90–95% of all bone cells) within bone tissue. Osteocytes are connected by long cell projections with osteoblasts and osteoclasts on the mineralized surfaces of bone and sense the signals, including hormones and mechanical stimuli, to initiate bone remodeling. Increased ROS due to estrogen deficiency cause osteocyte apoptosis, which contributes to osteoclastogenesis [267, 268]. Indeed, these apoptotic osteocytes are able to trigger bone resorption through osteoclastogenic RANKL and additionally inhibit Wnt/β-catenin signaling-induced bone formation by release of Dickkopf-related protein 1 and sclerostin release. Furthermore, osteocytes have also been demonstrated to regulate cellular senescence in bone and bone marrow cells in mice [269]. Therefore, oxidative stress and consequent osteocyte ablation is involved in altered mesenchymal lineage commitment, causing impairment of osteogenesis and induction of osteoclastogenesis. Moreover, osteocyte deficiency in mice results in mobilization of hematopoietic lineage cells and differentiation towards the myeloid lineage, with increased common myeloid precursor cells, neutrophils, and monocytes, all of which can participate in ROS generation in the aging bone. Besides excessive ROS production by diverse immune cells as well as old and damaged bone cells, it has been shown for both, osteocytes and osteoblasts that a deletion of mitochondrial antioxidative SOD2 activity leads to age-related OP, for example, via dysregulated RANKL expression [9, 270]. Additionally, the decline in estrogen, which is a well-known antioxidant, reducing, for example, peroxide production by mitochondria, can lead to a diminished antioxidant status and consequently cause decreased autophagy and increased apoptosis in osteocytes [227].

**Fig. 4:**
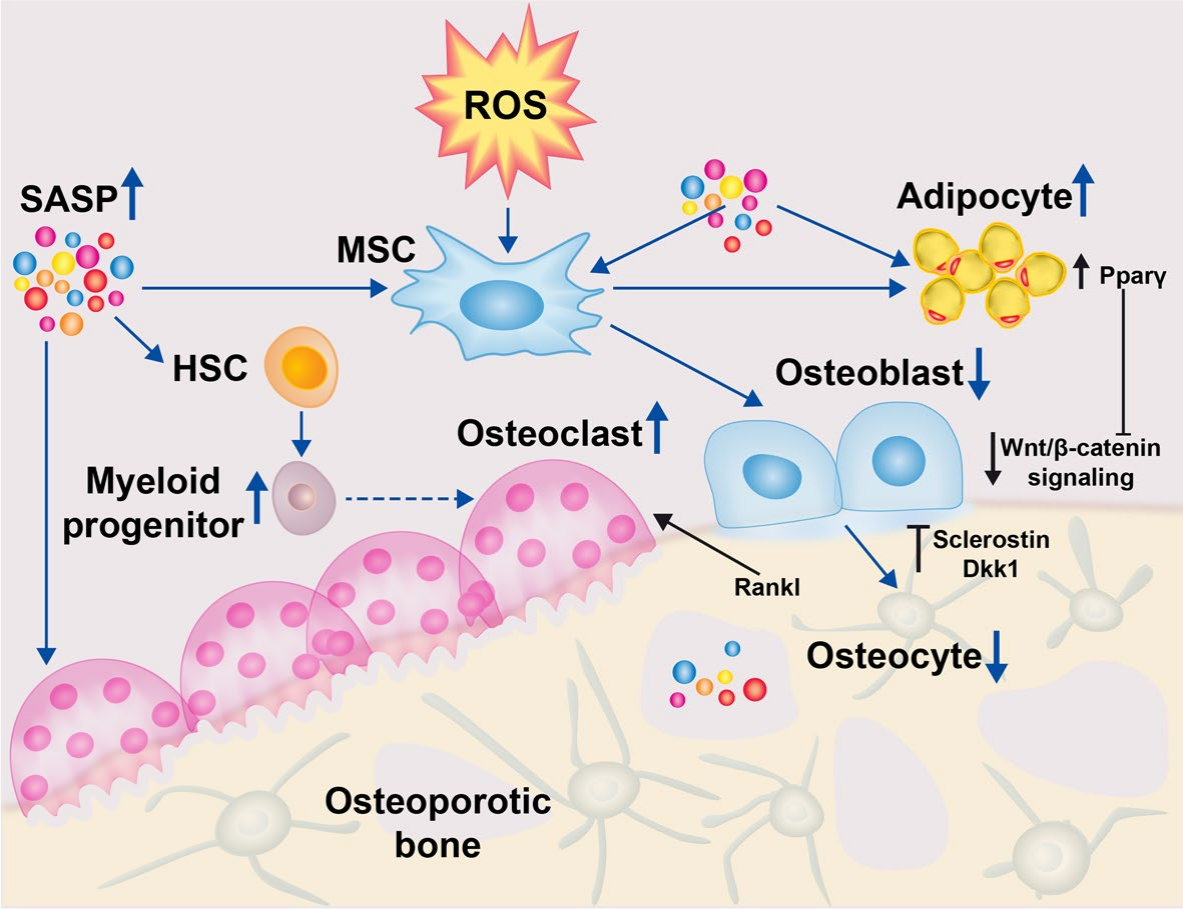
Role of ROS in bone cell fate regulation. ROS (reactive oxygen species) generation due to aging and estrogen deficiency is accompanied by increased cellular senescence characterized by the senescence-associated secretory phenotype (SASP) in the bone tissue. The SASP facilitates the accumulation of adipocytes in the bone marrow at the expense of osteoblast formation by inducing peroxisome proliferator activated receptor gamma (Pparγ) in mesenchymal progenitor cells (MSCs). Additionally, the SASP promotes myeloid progenitor development from the hematopoietic stem cell (HSC) lineage. Consequently, an elevated monocyte level is associated with an increase of osteoclast formation. Apoptotic osteocytes are involved in increased bone resorption by producing an unbalanced RANKL level. Moreover, apoptotic osteocytes induce sclerostin and Dickkopf-related protein (Dkk) 1 production, which inhibits Wnt/b-catenin mediated osteogenesis

The influence of ROS in cell fate regulation is schematically outlined in Fig. 4.

### Role of mitochondrial dysfunction and mitophagy in OP

Unbalanced ROS generation due to mitochondrial dysfunction has been demonstrated to be associated with OP [308]. Because mitochondria are the main source of aerobic energy production in the cell, they are involved in essential cell processes, including cell metabolism and differentiation [271]. However, inflammatory and metabolic diseases, such as OP, are associated with aberrant mitochondrial function and thus enhanced ROS production [272]. Several studies have demonstrated the consequences of oxidative stress on mitochondrial function, including impaired ATP synthesis, decreased mitochondrial membrane potential, and altered calcium homeostasis [273].

Mitochondrial dynamics accompany MSC fate commitment. Under physiologic conditions, mitochondria display a fragmented phenotype generated by mitochondrial shaping proteins, for example, fission and fusion proteins, and only a few deep invaginations in the cristae are localized around the nucleus of MSCs. By contrast, in mature osteoblasts and adipocytes, mitochondria form an interconnected, elongated network and exhibit higher cristae density. During MSC differentiation, mitochondrial biogenesis is activated and mitochondria are reorganized resulting in its distribution throughout the cytoplasm. Moreover, osteoblastogenesis and adipogenesis are associated with an increase in mitochondrial membrane potential, respiratory enzyme complexes, and oxygen consumption. Consequently, intracellular ATP generation in differentiating cells is primarily based upon oxidative phosphorylation and not on glycolysis, resulting in greater ATP but also ROS generation [274]. It has been demonstrated that MSCs are able to use mitochondrial transfer to rescue aerobic respiration and thereby modulate their cell metabolism. In response to ROS and DAMPs, including damaged mitochondria and mitochondrial products, such as mtDNA, MSCs use various transfer mechanisms to transfer intact mitochondria to injured cells [275]. Moreover, it was observed that macrophages are able to promote MSC osteogenic differentiation by mitochondrial transfer, implying that MSCs act as donor as well as recipient cells. However, under OP conditions, pro-inflammatory M1 macrophages transfer oxidatively damaged mitochondria to MSCs [276]. These damaged mitochondria affect intermediates of the tricarboxylic acid (TCA) cycle, including succinate, in the recipient MSCs. In agreement with this, succinate levels have been shown to be elevated in metabolic and inflammation-related diseases. This abnormal succinate accumulation in MSCs may be caused by decreased activity of oxidases, such as succinate dehydrogenase. Moreover, metabolic intermediates of the TCA cycle, such as succinate, have been demonstrated to be key regulators of various biological processes and able to stimulate osteoclastogenesis and inflammatory gene expression [276, 277]. For example, extracellular succinate is able to stimulate osteoclastogenesis by binding to its receptor on osteoclastogenic lineage cells. Additionally, age-related increased ROS result in osteoblast dysfunction by affecting MSC mitochondrial dynamics. Therefore, it has been shown that ROS overgeneration, for example, is able to promote mitochondrial fission and fragmentation and is associated with the dysregulation of mitochondrial dynamics proteins, including mitochondrial dynamin-related protein 1 and mitofusin 2 [278]. Consequently, oxidative stress can negatively affect mitochondrial function, leading to an impaired energy supply and resulting in osteoblast dysfunction.

Senile OP (type II OP) is commonly associated with senescence and decreased autophagic activity [279]. Autophagy is a highly conserved cellular process, whose main function is the lysosomal degradation and recycling of damaged intracellular components, such as organelles and proteins [280]. It greatly participates in cell metabolism under physiologic and pathologic conditions, thus contributing to bone cell homeostasis. Various studies demonstrated that autophagy is also involved in the regulation of osteoblast mineralization and osteoclast differentiation. Conditional knockdown of autophagy-related genes, such as autophagy-related gene (ATG) 7 and ATG5 in osteoblasts, causes impaired bone mineralization and increased osteoclastogenesis, respectively, leading to bone mass loss [280]. Besides “bulk” or “non-selective” autophagy, which indiscriminately secludes and degrades intracellular products, “selective” autophagy degrades specific targets and organelles, such as RNA, protein aggregates, or mitochondria [281]. Mitophagy, therefore, is a process by which damaged or dysfunctional mitochondria are selectively removed through autophagy, which represents a decisive mechanism in maintaining mitochondrial quality and cellular homeostasis. Malfunctioning of the respiratory chain complex proteins results in energy deficiency and ROS accumulation. Moreover, the release of mtDNA and other DAMPs from damaged mitochondria is thought to trigger inflammation and ROS generation, contributing to the pathogenesis of OP [280, 281]. Studies have shown that ROS-induced mitochondrial damage activates mitophagy in osteoblasts in order to remove damaged mitochondria and maintain mitochondrial function and cellular homeostasis. In this way, the cell can prevent excessive ROS production by dysfunctional mitochondria. However, ROS-generated AOPPs can cause more severe damage, thus inducing mitochondrial ROS in osteoblastic cells, exacerbating oxidative stress, and promoting apoptosis [282].

**Fig. 5:**
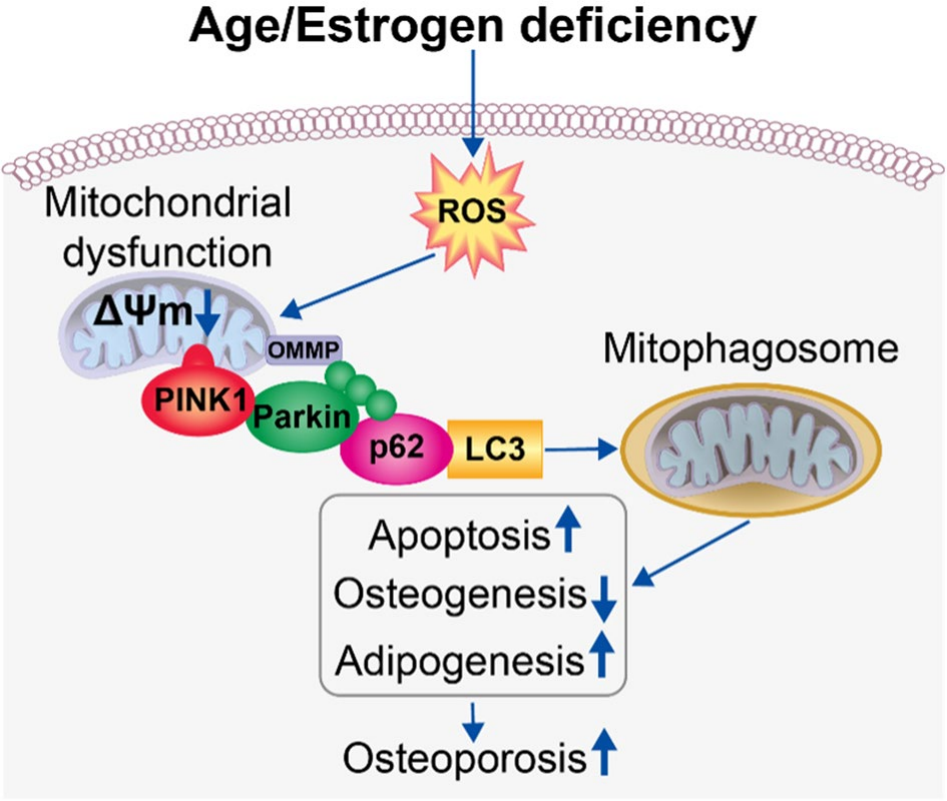
Role of ROS in regulating mitophagy in OP. Age and estrogen deficiency cause enhanced reactive oxygen species (ROS) generation in MSCs. ROS lead to mitochondrial dysfunction accompanied by a reduced mitochondrial membrane potential (ΔΨm). Subsequent PINK1 accumulation at the outer surface of the mitochondrial membrane initiates mitophagy by recruitment of Parkin that promotes ubiquitination (green dots) of OMM proteins (OMMP). Ubiquitinated proteins bind directly or indirectly to autophagy receptor LC3 via p62 on mitophagosomes. Aberrant mitophagy may result in increased apoptosis, promoting marrow adipogenesis as well as suppressing osteogenesis, all consequently contributing to OP development

Several signaling pathways involved in the regulation of mitophagy have been identified, with the Pink1/Parkin pathway being the best studied regulatory pathway [282]. Pink1 is a serine/threonine kinase possessing an N-terminal mitochondrial target sequence for localization at the mitochondrial surface, specifically at the OMM. Under physiologic conditions, Pink1 expression is increased during osteoblast differentiation to maintain mitochondrial homeostasis. Mitochondrial damage, initiated by increased ROS in aging bone or due to estrogen deficiency, enhances the presence of Pink on the mitochondrial surface in MSCs (Fig. 5). Therefore, the activity of Pink serves as a sensor of mitochondrial damage. The accumulated Pink1 subsequently recruits and activates the ubiquitin E3 ligase, Parkin, to amplify mitophagy signaling. Parkin ubiquitinates OMM proteins, such as voltage-dependent anion channel 1. These ubiquitinated OMM proteins bind to mitophagosomes either by direct binding to microtubule-associated protein 1A/1B-light chain 3 (LC3) II embedded in the membrane of autophagosomes, or indirectly through p62/Sqstm1. The latter contains an LC3-interacting domain, which enables binding to LC3. Binding of the ubiquitinated OMM proteins to the autophagy receptors p62/Sqstm1 and LC3 is necessary to initiate mitophagy (Fig. 5). In aging bone, as mentioned above, MSCs lose their differentiation potential towards the osteoblast lineage and, instead, differentiate into adipocytes, resulting in bone loss and fat accumulation in the bone marrow. Using an optineurin knockout mouse model,, the mitophagy receptor optineurin was previously shown to play a critical role in the cell fate decision of MSCs and the bone-fat balance by regulating fatty acid binding protein 3 degradation mediated by selective autophagy [283]. Furthermore, UNC51-like autophagy activating kinase 1 (ULK1), another adaptor protein associated with mitophagy, can interact indirectly with LC3 to induce mitophagy. Ulk1 gene knockout demonstrated the significant relevance of unimpaired mitophagy in maintaining bone homeostasis in a bone metastasis mouse model, because Ulk1 deletion caused an increased expression of osteoclast function-related genes, including carbonic anhydrase 2 and cathepsin k, and led to bone loss [284].

Sirt3-mediated mitophagy in MSCs has a protective effect against OP development. Therefore, it has been demonstrated that, for example, increased mitochondrial Sirt3 expression ameliorated MSC senescence caused by advanced glycation end products by promoting mitophagy in senile OP [32]. Mitophagy in osteoclasts also plays a crucial role in maintaining bone homeostasis. Therefore, ROS released from damaged mitochondria and blockade of mitophagy in macrophages has been shown to cause NLRP3 inflammasome activation, which generates inflammatory factors that stimulate osteoclastogenesis [285].

It is clear that manipulation of proteins that regulate and restore mitophagy may represent a therapeutic approach to prevent or ameliorate OP. For example, Pink1/Parkin-mediated mitophagy activated by rapamycin can significantly decrease osteoblast apoptosis by eliminating damaged mitochondria in a model of oxidative stress-induced osteoblastic cell dysfunction [286]. Besides rapamycin, metformin has been shown to diminish ROS generation in MSCs and to enhance osteogenesis and bone formation in mice by promoting autophagy [287]. Moreover, mitophagy upregulation is able to diminish the plasma AOPP concentration in mice, thus inhibiting osteoblast apoptosis and bone loss induced by the accumulation of these harmful oxidative stress products [282].

## Oxidative stress and mitochondrial dysfunction as therapeutic targets in OA and OP

As described in the sections above, oxidative stress and mitochondrial dysfunction can adversely affect cellular behavior and fate, thus contributing to OA and OP pathogeneses. Therefore, both, cellular antioxidant defense mechanisms and mitochondria represent promising and longstanding therapeutic targets to circumvent harmful consequences of ROS on cartilage and bone tissue. In addition to a myriad of natural antioxidants, including alkaloids, flavonoids, phenols, and terpenoids, which have been extensively discussed in previous reviews [227, 288], new small molecules and peptides possessing direct or indirect antioxidative features are of considerable interest. SS-31 (elamipretide), for example, is a unique small peptide, which stabilizes the inner mitochondrial membrane by specific binding to the phospholipid cardiolipin. In this way, SS-31 prevents ROS-mediated cardiolipin peroxidation, maintains mitochondria cristae structure, and supports efficient ATP production, while preventing apoptosis by promotion of the cytochrome c-cardiolipin interaction [142, 143]. MDL-800 and SRT1720, by contrast, are selective sirtuin activators for SIRT6 and SIRT1, respectively. Binding of MDL-800 or SRT1720 to the allosteric site of these deacetylases results in an increased affinity to their substrates, thus enhancing their enzyme activity [146, 221]. Interestingly, resveratrol was also demonstrated to act as a sirtuin activator by inducing the AMPK pathway and subsequently elevating intracellular levels of the enzyme’s cofactor NAD^+^ [289].

There are various therapeutic strategies and antioxidant agents to pharmacologically address oxidative stress and mitochondrial dysfunction in OA and OP. Table 1 summarizes therapeutic agents of different classes, which have mostly been described in the sections above, and provides a short overview of the respective therapeutic targets and effects.

**Table 1:** Overview of different therapeutic agents, the individual therapeutic targets, and effects

Therapeutic agents	Class	Therapeutic target	Therapeutic effects	References
SS-31 (elamipretide, MTP-131)	Small peptide, mitoprotectin	Mitochondria, cardiolipin	Stabilization of MIM via binding to cardiolipin; cell protection (prevents cytochrome c release); maintenance of mitochondrial function (mitoprotection)	[142, 143]
Omaveloxolone	Nrf2 activator	Nrf2/ARE-pathway	Upregulation of antioxidant target genes, e.g., HO-1 and SOD2; cell protection (anti-apoptotic response); maintenance of tissue homeostasis	[144, 290]
Theaflavin-3,3′-digallate	Natural phenol (black tea), antioxidant	Nrf2/ARE-pathway	Upregulation of antioxidant targets, e.g., GPX4 and HO-1; prevention of ferroptosis (upregulation of FTH-1, SLC7A11, and GPX4); maintenance of tissue homeostasis	[155]
Deferoxamine	Iron chelator	Nrf2/ARE-pathway, HIF-1a	Upregulation of antioxidant targets, e.g., HO-1, and NQO-1; prevention of ferroptosis (reduction of ACSL4, LOX15, P53, and LPCAT3); stabilization of HIF-1a, increase of osteogenic differentiation/ improvement of fracture healing	[154, 291]
Licochalcone A	Natural phenol (Glycyrrhiza roots), antioxidant	Nrf2/ARE-pathway; NFκB pathway	Inhibition of NFκB pathway; prevention of LPS-induced pyroptosis (reduction of NLRP3, GSDMD, caspase‐1, IL‐1β, and IL‐18); upregulation of antioxidant targets, e.g., Nrf2 and HO-1,	[176]
Pioglitazone	Antidiabetic drug (thiazolidinedione)	Nrf2/ARE-pathway, mitochondria (via PGC1-α/ Δψm pathway)	Activation of Nrf2- and PGC1-α/Δψm pathway, Prevention of LPS/ATP-induced pyroptosis (downregulation of NLRP3, caspase-1, IL‐1β, IL‐18, and GSDMD-N); increase of mitochondrial function and biogenesis (lower ROS production)	[177]
SRT1720	SIRT1 activator	SIRT1	Activation of autophagy; maintenance of tissue homeostasis	[146, 292]
NAC	Antioxidant	ROS (as direct scavenger and precursor of glutathione)	Cell protection (prevents apoptosis and necroptosis); downregulation of redox-sensitive signaling; maintenance of tissue homeostasis	[142, 146]
Simvastatin	Statin, Senomorphic	HMG-CoA	Antioxidative properties (upregulation of SOD2, suppression of NOX2, NOX4); increase of osteogenesis; maintenance of tissue homeostasis	[293, 294]
Resveratrol	Natural phenolic compound, Senomorphic	SIRT1 Mitochondria (mitophagy) via mTOR/AMPK pathway	Induction of antioxidant defense by HO-1 and Nrf2; reduction of iNOS and NO, induction of autophagy, suppression of NFkB signaling pathways; maintenance of tissue homeostasis	[220, 289]
Urolithin A	Natural postbiotic compound	Mitochondria (mitophagy) Nrf2-pathway	Improvement of mitochondrial function (enhances mitophagy); induction of Nrf2 and thus antioxidative proteins; maintenance of tissue homeostasis	[217]
MDL-800	Allosteric activator of SIRT6	SIRT6	Attenuation of ROS activity and downregulation of senescence markers; maintenance of tissue homeostasis	[221]
Metformin	Antidiabetic drug (biguanide), AMPK agonist	Mitochondria/ mitophagy (via mTOR/AMPK pathway)	Reduction of ROS production and senescence; Increase of osteogenesis and autophagy; maintenance of tissue homeostasis	[23, 295]
Rapamycin (Sirolimus)	Makrolide (macrocyclic lacton)	Mitochondria/ mitophagy (via mTOR/AMPK pathway)	Increase of PINK1/Parkin-dependent mitophagy; inhibition of apoptosis; maintenance of tissue homeostasis	[282]

*ACSL4* acyl-CoA synthetase long-chain family member 4, *AMPK* AMP-activated protein kinase, *FH-1* ferritin heavy chain 1, *HMG-CoA* hydroxymethylglutaryl-coenzyme A, *iNOS* inducible NO synthase, *LOX15* 15-lipoxygenase, *LPCAT3* lysophosphatidylcholine acyltransferase 3, *MIM* mitochondrial inner membrane, *NQO-1* NAD(P)H dehydrogenase (quinone 1), *PGC-1* PPAR-γ coactivator-1, *SLC7A11* light chain subunit of the cystine/glutamate anticarrier

## Conclusions

Mitochondrial dysfunction and oxidative stress do indeed play a decisive role in cell fate decision. Whether ROS has beneficial or harmful consequences depends on several factors. Under physiologic conditions, ROS are essential regulators of developmental and regenerative processes, such as stem cell differentiation, and constitute an inherent component of cellular communication. However, excessive ROS accumulation contributes to a pathophysiologic environment and can have an impairing effect on tissue repair and homeostasis. The same applies for ROS-induced cell death and senescence, which firstly may appear to represent detrimental processes, but can also contribute to tissue regeneration and health.

Overall, the consequences of oxidative stress strongly depend on the cellular antioxidant defense status, which is closely associated with age and life style. While a healthy cell can readily cope with enhanced ROS levels, a cell exposed to several stress factors, comprising pro-inflammatory mediators and DAMPs, might be unable to circumvent irreversible macromolecular damage. This also applies in tissues in which transiently occurring senescent cells are normally eliminated by phagocytotic immune cells, while a defective clearance due to aging or general alterations in immune cell response results in chronic senescence and subsequent degeneration.

Taken together, mitochondrial dysfunction and oxidative stress represent a major target in age-related and degenerative diseases as we here illustrated with the example of OA and OP. By increasing the cellular antioxidative defense system or reducing mitochondrial ROS production, harmful consequences of excessive ROS levels can be prevented, allowing the maintenance of physiologic processes and regeneration to proceed.

## Data Availability

Not applicable.

## Abbreviations

4-HNE: 4-Hydroxynonenal
8-OHdG: 8-Hydroxy 2′-deoxyguanosine
ADAMTS: A desintegrin metalloproteinases with thrombospontin motive
AIF: Apoptosis inducing factor
AMP: Adenosine monophosphate
AMPK: AMP-activated protein kinase
AOPPs: Advanced oxidation protein products
AP-1: Activation protein 1
ARE: Antioxidant response element
ATM: Ataxia Telangiectasia Mutated
ATP: Adenosine triphosphate
ATR: Ataxia telangiectasia and Rad3 related
BMAT: Bone marrow adipose tissue
BMD: Bone mineral destiny
BNIP3: Bcl-2/adenovirus E1B 19-kDa interacting protein
CAT: Catalase
CARD: Caspase recruitment domain
CD: Cluster of differentiation
CXCL1: C-X-C Motif Chemokine Ligand 1
DAMPs: Damage-associated molecular patterns
DNA: Desoxyribonucleic acid
Erk1/2: Extracellular signal-regulated kinase 1/2
FoxO: Forkhead box O
GPX: Glutathione peroxidase
GSDMD: Gasdermins
GSH: Glutathione
GSSG: Glutathione disulfide
H_2_O_2_: Hydrogen peroxide
HIF-1/2a: Hypoxia-inducible factor 1/2a
HO-1: Heme oxygenase-1
IL: Interleukin
JNK: C-Jun N-terminal kinases
Keap1: Kelch-like ECH-associated protein 1
M-CSF: Monocyte/macrophage colony-stimulating factor
MAPK: Mitogen-activated protein kinase
MDA: Malondialdehyde
Mfn2: Mitofusin 2
MKK: Mitogen-activated protein kinase-kinase
MLKL: Mixed lineage kinase domain-like
MMPs: Matrix metalloproteinases
MSC: Mesenchymal stem cells
mtDNA: Mitochondrial DNA
NAD(PH): Nicotinamide adenine dinucleotide (phosphate)
NF-κB: Nuclear factor-κB
NLRP3: Nucleotide-binding domain and leucine-rich repeat protein-3
NO: Nitric oxide
NOX: NADPH oxidase
Nrf2: Nuclear factor erythroid 2-related factor 2
O2^−^: Superoxide
OA: Osteoarthritis
·OH: Hydroxyl radical
OMM: Outer mitochondrial membrane
ONOO^−^: Peroxynitrite
PINK1: PTEN-induced putative kinase
PPARγ: Peroxisome proliferator-activated receptor γ
PTOA: Posttraumatic osteoarthritis
RANK(L): Receptor activator of nuclear factor kappa-B (ligand)
RIPK: Receptor-interacting serine/threonine-protein kinase
ROS/RNS: Reactive oxygen/ nitrogen species
SA-β-Gal: Senescence-associated β-galactosidase
SASP: Senescence-associated secretory phenotype
SIPS: Stress-induced premature senescence
SIRT: Sirtuin
SOD: Superoxide dismutase
SQSTM1: Autophagic adaptor protein sequestosome-1
Th17: Inflammatory T cells
TNF(R1): Tumor necrosis factor (receptor 1)
